# Prediction of iatrogenic preterm birth in patients with scarred uterus: a retrospective cohort study in Northeast China

**DOI:** 10.1186/s12884-020-03165-7

**Published:** 2020-08-26

**Authors:** Liyang Zhang, Hongtian Li, Jiapo Li, Yue Hou, Buxuan Xu, Na Li, Tian Yang, Caixia Liu, Chong Qiao

**Affiliations:** 1 Department of Obstetrics and Gynecology, Shengjing Hospital, China Medical University, Shenyang, Liaoning Province China; 2 Institute of Reproductive and Child Health/National Health Commission Key Laboratory of Reproductive Health, Peking University Health Science Center, No. 38 Xueyuan Rd, Beijing, 100191 China; 3 China Medical University, Shenyang, Liaoning Province China

**Keywords:** Iatrogenic preterm birth, Scarred uterus, Prediction model

## Abstract

**Background:**

To build a novel and simple model to predict iatrogenic preterm birth in pregnant women with scarred uteri.

**Methods:**

In this retrospective, observational, single-centre cohort study, data from 2315 patients with scarred uteri were collected. Multiple logistic regression analysis and mathematical modelling were used to develop a risk evaluation tool for iatrogenic preterm birth. After modelling, the calibration and discrimination of the model along with decision curve analysis were checked and performed to ensure clinical applicability.

**Results:**

Among the 2315 patients, 417 (18.0%) had iatrogenic preterm births. The following variables were included in the model: interpregnancy interval (0 to < 12 months, OR 5.33 (95% Cl 1.79–15.91), *P* = 0.003; 13 to < 24 months (reference), 25 to < 60 months, OR 1.80 (95% CI 0.96–3.40), *P* = 0.068; ≥ 60 months, OR 1.60 (95% Cl 0.86–2.97), *P* = 0.14), height (OR 0.95, (95% CI 0.92–0.98), *P* = 0.003), parity (parity ≤1 (reference), parity = 2, OR 2.92 (95% CI 1.71–4.96), *P* < 0.0001; parity ≥3, OR 8.26, (95% CI 2.29–29.76), *P* = 0.001), number of vaginal bleeding (OR 1.81, (95% Cl 1.36–2.41), *P* < 0.0001), hypertension in pregnancy (OR 9.52 (95% CI 6.46–14.03), *P* < 0.0001), and placenta previa (OR 4.21, (95% CI 2.85–6.22), *P* < 0.0001). Finally, a nomogram was developed.

**Conclusions:**

In this study, we built a model to predict iatrogenic preterm birth for pregnant women with scarred uteri. The nomogram we created can assist doctors in evaluating the risk of iatrogenic preterm birth and help in making referrals; thus, better medical care can be given to improve the prognosis of patients and foetuses.

## Background

Preterm birth (PTB) remains a major unsolved problem in modern obstetrics and is significantly associated with infant mortality, long-term morbidity and neurodevelopmental impairment. The prevalence of preterm birth is 5–18% of all live births worldwide [1]. To date, many studies have been performed regarding the prediction of preterm birth. The risk factors include an obstetric history of previous adverse events, maternal age [2], socio-economic factors [3], maternal obesity [4], placenta previa [5], multiple gestations [6], cervical length [7], and other biomarkers [8–11]. However, most studies have been specifically restricted to spontaneous preterm birth. Over the past 24 years, spontaneous preterm birth has declined by 25%, while the incidence of iatrogenic preterm birth has increased, representing nearly 30% of all preterm births [12]. Efforts to study iatrogenic preterm birth have been ignored.

China has one of the highest caesarean section rates in the world. With new family planning policies emerging, an increasing number of women have decided to have a second child, which makes pregnancy with a scarred uterus an increasingly prominent problem. Pregnancy with a scarred uterus has been considered risky and closely related to adverse pregnancy outcomes [13]. Those who end up with iatrogenic preterm birth are at much higher risk of having the pregnancy terminated if indications allow. At present, TOLAC (trial of labour after caesarean) is not a common practice in China; most patients with scarred uteri have the pregnancy terminated by caesarean section. Operation techniques and medical levels in rural areas still lag those in urban areas. In the hierarchical medical system of China, the evaluation of high-risk patients is not precise; referral is not timely, and incorrect referral frequently occurs. Patients who will ultimately have iatrogenic preterm birth often fail to receive good medical care. Thus, we developed a novel, simple prediction model of iatrogenic preterm birth for pregnant women with scarred uteri using a Chinese patient database to precisely evaluate the risk of preterm birth and help in making referrals, which will further benefit the prognosis of pregnant women and foetuses.

## Methods

### Study design and participants

This study included data from a large retrospective cohort study in Northeast China from 2014 to 2017 at Shengjing Hospital, a regional tertiary medical centre. The study was approved by the local ethics committee (ethics committee of China Medical University) in Shenyang. The original cohort study recruited 8697 patients with scarred uterus from all 69,931 deliveries made during the study period to evaluate the impact of scarred uterus on the subsequent pregnancies. The inclusion criteria for this study were all women with a scarred uterus from caesarean section or myomectomy AND singleton pregnancies at gestational week 20 or beyond. (including cases of stillbirth). The patients were excluded if they had severe systematic disease, had a twin or greater pregnancy, were unable to speak Chinese, or had no access to a telephone. Finally, 2315 patients remained after exclusion.

### Variable assessments

Preterm deliveries are those that occur at less than 37 weeks of gestational age. Our study recruited pregnant women who went into labour between the 20th and 42nd weeks of gestation. Diagnostic records and actual labour weeks were checked to determine whether the patient had iatrogenic preterm birth. Patients with spontaneous preterm birth were also included in this study and treated as controls. A patient could start labour before 37 weeks of gestation and finish with CS due to maternal or foetal medical concerns; cases such as this were still considered spontaneous preterm births. Delivery of the baby according to indications and decided by obstetricians was considered an iatrogenic preterm birth. Methods of termination included CS and TOLAC.

The other variables included in our study were as follows: maternal age, height, weight before delivery, parity, interpregnancy interval (IPI), number of vaginal bleeding during the pregnancy, foetal position, myomectomy, dysmenorrhea, regularity of prenatal examination, conception method, placenta previa, hypertension during pregnancy, and gestational diabetes mellitus. These data were all collected from a computerized medical record system including all data concerning basic individual information, medical and obstetric histories, and pregnancy outcomes. Both maternal age and height were collected at the first prenatal visit. Regarding weight, the data collected in our study are the weights before delivery because of the loss of data on weight at the first prenatal visit. Hypertension during pregnancy was defined according to the American College of Obstetrics and Gynecology (ACOG) criteria [14]. Placenta previa was defined as a placenta overlying the internal cervical os [15]. Gestational diabetes mellitus (GDM) was defined as “the type of glucose intolerance that develops in the second and third trimesters of pregnancy, resulting in hyperglycemia of variable severity” [16] and was diagnosed used an oral glucose tolerance test (OGTT) between 24 and 28 weeks gestation by the International Association of the Diabetes and Pregnancy Study Groups (IADPSG) criteria. Number of vaginal bleeding during pregnancy were self-reported and defined as the total number of vaginal bleeding experiences during the whole pregnancy.

### Establishment of the model

A total of 2315 cases were randomly split into a training (*n* = 1566) and validation set (*n* = 749). Distributions of continuous variables were assessed for normality using the Kolmogorov-Smirnov test; none of the continuous variables were normally distributed in this study. Categorical variables are presented as percentages, and continuous variables are presented as the median (25 and 75% quantiles). Bivariate analyses were performed by the Mann-Whitney U test or Fisher’s exact test.

Simple and multiple logistic regression analyses were used to model risk factors for preterm birth. The interaction between variables may lead to differences in the results of univariable and multivariable analysis. To avoid missing important risk factors, variables with *P* < 0.2 in the univariable analysis were included in the multivariable regression models using a forward stepwise algorithm. Finally, 6 variables were included in our model: height, parity, number of vaginal bleeding during pregnancy, IPI, placenta previa, and hypertension during pregnancy. In addition, odds ratios (ORs) and 95% confidence intervals (CIs) were calculated. The level of significance for the *P* value was set as 0.05.

Other important assessments are outlined here. Discriminative ability was assessed using the AUC c-statistic. The Hosmer-Lemeshow test was used to determine the adequacy of calibration, and a calibration plot was drawn. To evaluate multicollinearity, the variance inflation factor, tolerance, eigenvalue, and condition index were checked. Decision curve analysis was used to determine the clinical practicability. Finally, a nomogram was developed.

### Statistical analysis

SPSS version 25.0 (IBM Corp, Armonk, NY, USA) was used for statistical modelling. STATA Release 12 Software (StataCorp, College Station, TX) was used to perform the Hosmer-Lemeshow test and decision curve analysis. R (R Core Development Team) version 3.1.1 was used to develop calibration tests and the nomogram.

## Results

### Baseline characteristics

Of the 2315 enrolled patients, 417 (18.0%) had an iatrogenic preterm birth, and 160 (6.9%) had a spontaneous preterm birth. The median age was 32 (IQR 30–35) years. The median height was 162 (IQR 160–165) cm. The majority of those enrolled had not experienced vaginal bleeding during pregnancy (*n* = 2057, 88.9%) and had less than 2 periods of labour (*n* = 2171, 93.8%). Over half (*n* = 1212, 52.4%) of the patients had an IPI of more than 60 months, and the fewest proportion of patients hand an IPI of 0 to < 12 months (*n* = 35, 1.5%). Hypertension in pregnancy was reported for 9.1% of the patients, while placenta previa was present in 14.1% of all patients. Further details of the population characteristics are shown in Table 1.

**Table 1:** Characteristics of patients with or without iatrogenic preterm birth and univariable analysis in both datasets

Characteristic	Training Dataset			Validation Dataset	
	IPTB = 0(*n* = 1283)	IPTB = 1(*n* = 283)	*P*	IPTB = 0(*n* = 615)	IPTB = 1(*n* = 134)
**Age, No. (%)**			0.866*		
≤ 35	991 (82.04)	217 (17.96)		477 (82.10)	104 (17.90)
>35	292 (81.56)	66 (18.44)		318 (82.14)	30 (17.86)
**Parity, No. (%)**					
≤1 (reference)	1219 (83.04)	249 (16.96)		585 (83.21)	118 (16.79)
2	59 (67.82)	28 (32.18)	< 0.0001*	28 (65.12)	15 (34.88)
≥ 3	5 (45.45)	6 (54.55)	0.004*	2 (66.67)	1 (33.3)
**Myomectomy, No. (%)**			0.866		
No	1249 (81.90)	276 (18.10)		598 (81.98)	132 (18.02)
Yes	34 (82.93)	7 (17.07)		17 (89.47)	2 (10.53)
**Dysmenorrhea, No. (%)**			0.583		
No	950 (81.62)	214 (18.38)		463 (82.53)	98 (17.47)
Yes	333 (82.84)	69 (17.16)		152 (80.85)	36 (19.15)
**IPI, No. (%)**					
13to < 24 months (reference)	133 (90.48)	14 (9.52)	0.002*	54 (85.71)	9 (14.29)
0 to < 12 months	15 (65.22)	8 (34.78)		9 (75.00)	3 (25.00)
25 to < 60 months	489 (83.02)	100 (16.98)	0.028*	234 (86.99)	35 (13.01)
≥ 60 months	646 (80.05)	161 (19.95)	0.003*	318 (78.52)	87 (21.48)
**Conception method, No.(%)**			0.746		
Normal	1276 (81.95)	281 (18.05)		614 (82.31)	132 (17.69)
IVF-ET	7 (77.78)	2 (22.22)		1 (33.33)	2 (66.67)
**number of vaginal bleeding during pregnancy, No. (%)**			< 0.0001*		
0	1182 (84.13)	223 (15.87)		559 (85.74)	93 (14.26)
1	85 (70.83)	35 (29.17)		44 (66.67)	22 (33.33)
2	13 (44.83)	16 (55.17)		9 (40.91)	13 (59.09)
≥ 3	3 (25.00)	9 (75.00)		3 (33.33)	6 (66.67)
**Regularity of prenatal examination, No. (%)**			0.024*		
Yes	984 (83.18)	199 (16.82)		470 (82.75)	98 (17.25)
No	299 (78.07)	84 (21.93)		145 (80.11)	36 (19.89)
**Foetal position, No. (%)**					
Cephalic presentation (reference)	1200 (83.22)	242 (16.78)		576 (83.24)	116 (16.76)
Breech presentation	68 (68.69)	31 (31.31)	< 0.0001*	30 (83.33)	6 (16.67)
Transverse presentation	15 (60.00)	10 (40.00)	0.004*	9 (42.86)	12 (57.14)
**GDM, No. (%)**			0.412		
No	1034 (82.32)	222 (17.68)		530 (82.94)	109 (17.06)
Yes	249 (80.32)	61 (19.68)		85 (77.27)	25 (22.73)
**Hypertension in pregnancy, No. (%)**			< 0.0001*		
No	1215 (85.56)	205 (14.44)		581 (84.94)	103 (15.06)
Yes	68 (46.58)	78 (53.42)		34 (52.31)	31 (47.69)
**Placental previa, No. (%)**			< 0.0001*		
No	1159 (85.54)	196 (14.46)		556 (87.70)	78 (12.30)
Yes	124 (58.77)	87 (41.23)		59 (51.30)	56 (48.70)
**Height (interquartile range)**	162 (160,165)	161 (160,165)	0.018*	162 (160.165)	162 (160.165)
**Weight (interquartile range)**	72 (66,79)	70 (65,80)	0.585	71 (65,78)	70 (65,78)

*Abbreviations*: *IPI* interpregnancy interval, *IPTB* iatrogenic preterm birth, *GDM* gestational diabetes mellitus

### Maternal and foetal indications of iatrogenic preterm birth

**Fig. 1:**
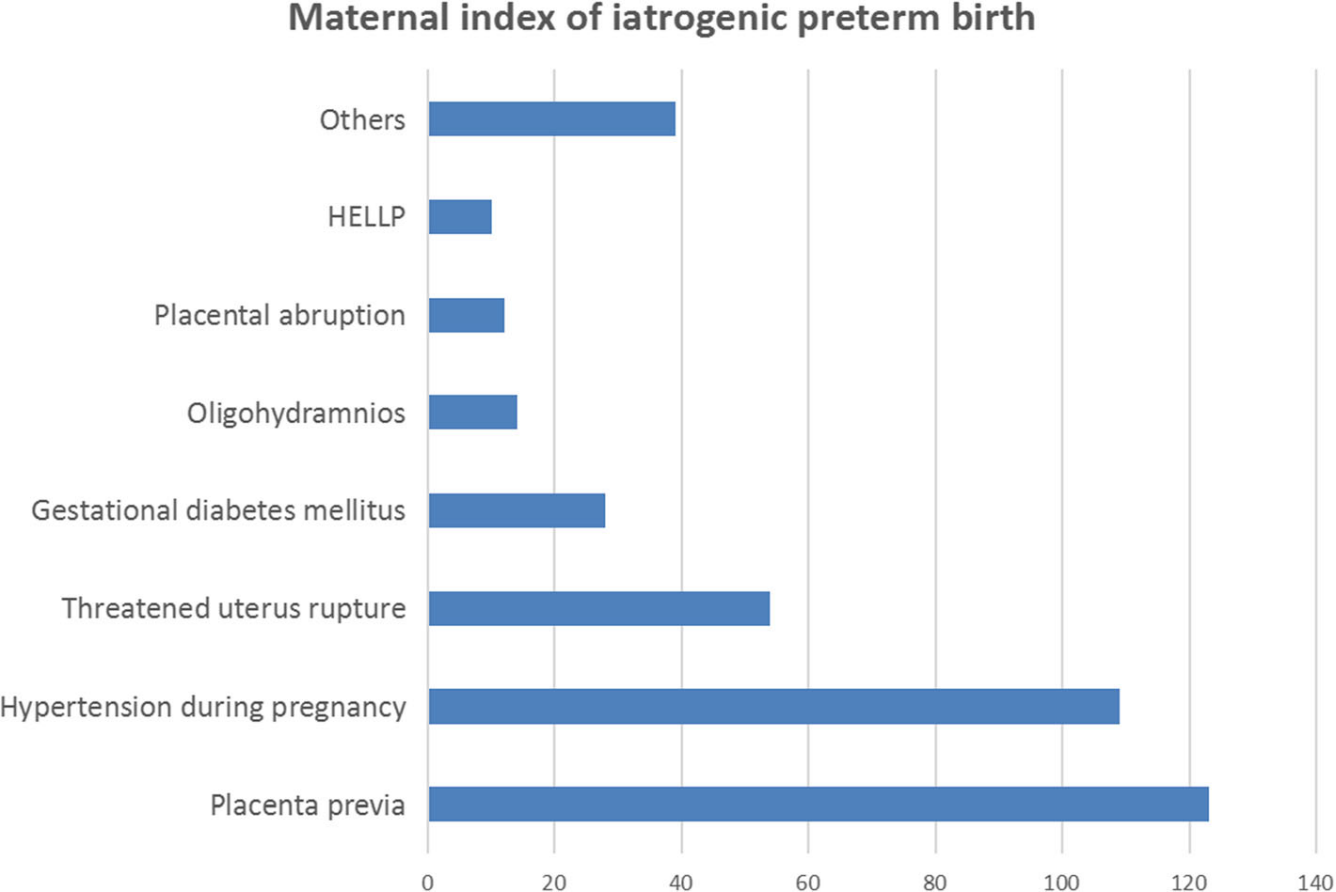
Maternal indications of IPTB

Among the 417 patients who had iatrogenic preterm birth, 318 (76.3%) were attributed to maternal reasons, 43 (10.3%) were attributed to foetal reasons, and the remaining 56 (13.4%) were associated with both maternal and foetal reasons. Among the maternal indications, placenta previa and hypertension during pregnancy accounted were the most common reasons for iatrogenic preterm birth. Other common indications included a threat of uterine rupture, GDM, and oligohydramnios. The maternal index of iatrogenic preterm birth (IPTB) is shown in Fig. 1. Meanwhile, the most common foetal indications were foetal distress (*n* = 41, 41.4%) and abnormal foetal position (*n* = 15, 15.2%).

### Multiple logistic regression model

After multivariable analysis, parity, interpregnancy interval, hypertension in pregnancy, placenta previa, height and number of vaginal bleeding during pregnancy were included in the final model. Among these factors, patients with hypertension during pregnancy had the highest risk of iatrogenic preterm birth (*OR* = 9.52, 95% CI 6.46–14.03), followed by a parity of more than 2 (*OR* = 8.26, 95% CI 2.29–29.76) and an interpregnancy interval of 0 to < 12 months (*OR* = 5.33, 95% CI 1.79–15.91). Increased height could protect patients from iatrogenic preterm birth. Further details are shown in Table 2. The VIF (variance inflation factor) of all the variables was approximately equal to 1 in our study, indicating low multicollinearity in this model.

**Table 2:** Qualified risk factors for preterm birth in the multiple logistic regression model

Variables	β	P	OR	95% CI
Parity				
≤1 (reference)				
2	1.070	< 0.0001	2.92	1.71–4.96
≥ 3	2.112	0.001	8.26	2.29–29.76
Interpregnancy interval				
13 to < 24 months (reference)				
0 to < 12 months	1.674	0.003	5.33	1.79–15.91
25 to < 60 months	0.590	0.068	1.80	0.96–3.40
≥ 60 months	0.467	0.140	1.60	0.86–2.97
Hypertension in pregnancy				
No (reference)				
Yes	2.253	< 0.0001	9.52	6.46–14.03
Placenta previa				
No (reference)				
Yes	1.438	< 0.0001	4.21	2.85–6.22
Height	−0.049	0.003	0.95	0.92–0.98
number of vaginal bleeding	0.593	< 0.0001	1.81	1.36–2.41

Note: OR, odds ratio; 95% CI, 95% confidence intervals

### Discrimination and calibration of the model

**Fig. 2:**
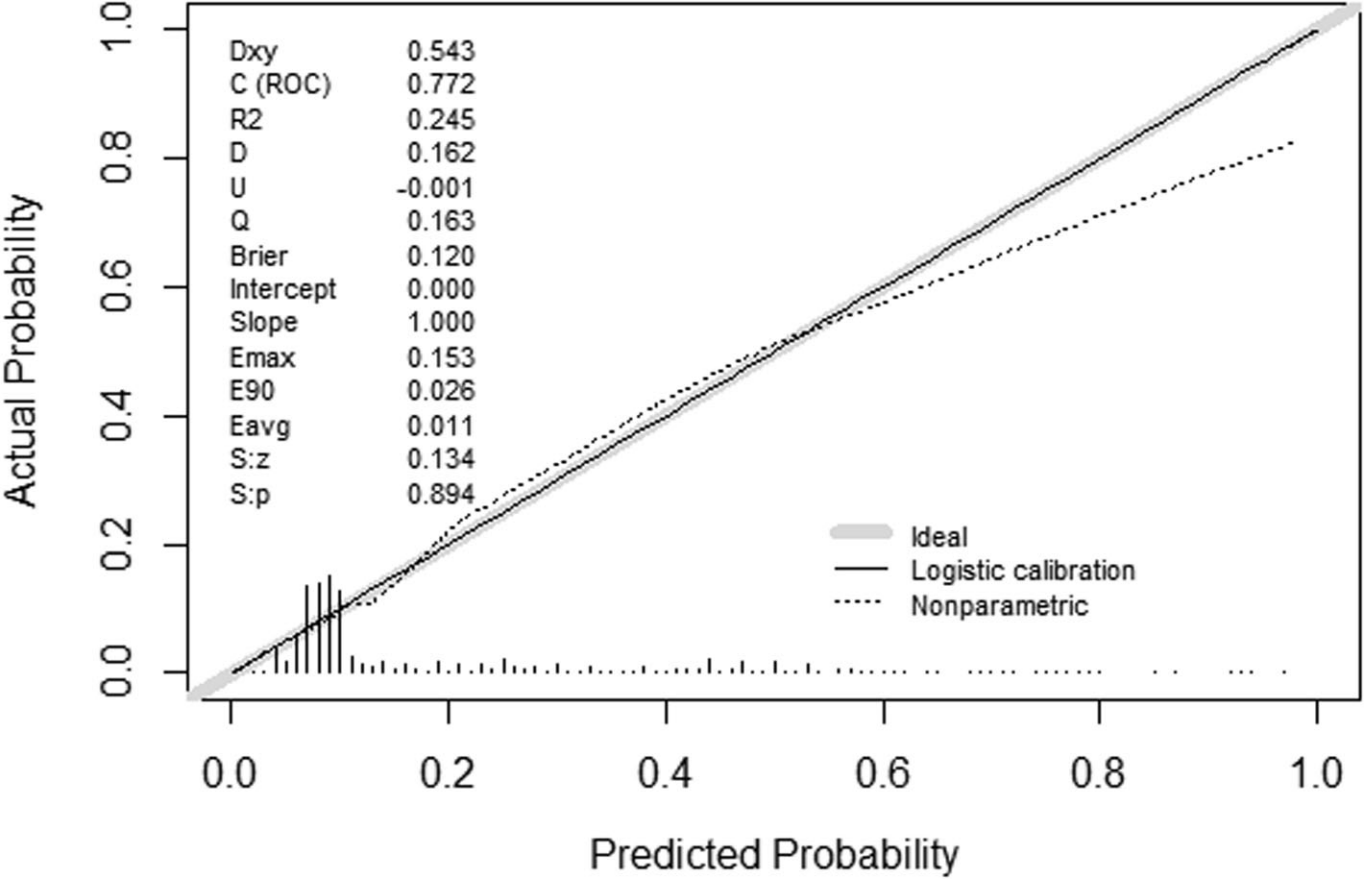
Calibration plot of the model

**Fig. 3:**
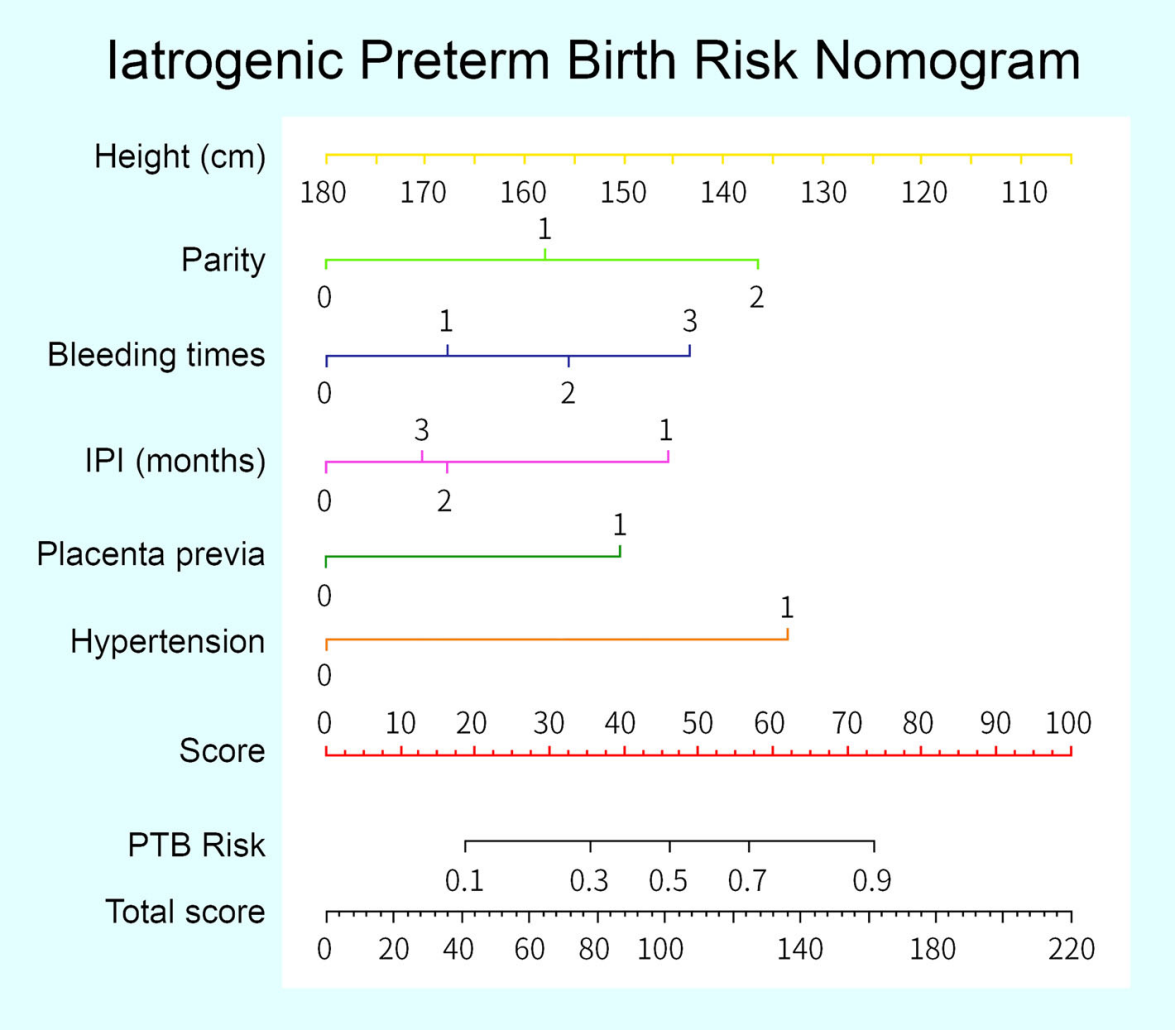
IPTB risk nomogram. Legend: Each predictor is assigned a score on each axis. The sum of all points for all predictors is computed and denoted as the total score. The risk of IPTB for the total score was converted to a probability of GDM

As mentioned in the Materials and Methods section, discrimination of the model was assessed by the AUC c-statistic. The AUC was 0.772 (95% Cl 0.739–0.804) in the training set and 0.776 (95% Cl 0.728–0.823) in the whole validation set. The suggested cutoff for this model was 0.15, and the false positive and false negative rates for the given cutoff were 29.1 and 21.6%, respectively. The calibration plots for the two sets are shown in Fig. 2. The training set and validation set were well calibrated. The *p* values from the Hosmer-Lemeshow test were 0.597 and 0.907 in the training and validation sets, respectively. Overall, the model showed good discrimination and calibration for both sets, and a nomogram was developed (Fig. 3).

### Decision curve analysis

**Fig. 4:**
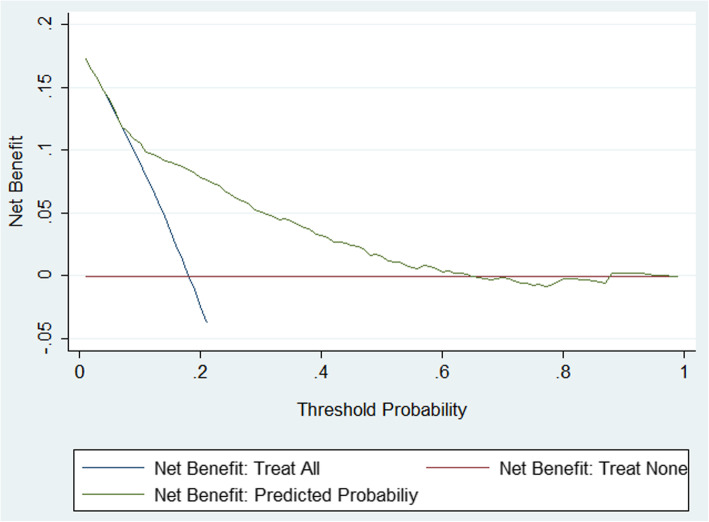
Decision curve analysis for IPTB. Legend: The decision curve analysis shows that if the threshold is between 0.15–0.6, use of the nomogram in this study to predict IPTB adds more benefit than either a treat-all-patients scheme or a treat-none scheme

Finally, to justify the clinical usefulness of the model, we assessed whether nomogram-assisted decisions would improve patient outcomes by performing decision curve analysis. Decision curves can help us calculate the net benefit of the use of our nomogram. The results of the decision curve analysis are shown in Fig. 4. The decision curve indicates that if the threshold probability for the patient or doctor is between 15 and 60%, the use of our nomogram to predict iatrogenic preterm birth adds more benefit than either a treat-all-patients scheme or a treat-none scheme.

## Discussion

In this study, six parameters were selected discreetly in the estimation of the overall risk of iatrogenic preterm birth: shorter maternal height, extremely low or advanced IPI, greater vaginal number of vaginal bleeding during pregnancy, higher parity, hypertension during pregnancy and placenta previa. By combining these factors, the risk of iatrogenic preterm birth can be well predicted.

Many studies have attempted to establish a simple way to predict preterm birth, but most focused on spontaneous preterm birth. In our study, many variables were used to predict iatrogenic preterm birth. Most of the underlying variables have been previously reported to impact preterm birth, while myomectomy and dysmenorrhea were included tentatively to assess their relationship with iatrogenic preterm birth. Maternal and foetal indications are the direct reasons for obstetricians to consider a patient at high risk of iatrogenic preterm birth or to tend to terminate the pregnancy. Severe complications during pregnancy, such as uterine rupture, are likely to lead to iatrogenic preterm birth. Although the prevalence of such complications is usually low, quick treatment is needed for these complications. Meanwhile, common foetal reasons for iatrogenic preterm birth, such as foetal distress, also require quick treatment given their sudden occurrence. Therefore, we mainly focused on chronic pregnancy complications, such as placenta previa and GDM, when we included complications into the model.

A shorter height has been associated with a progressive increase in the odds of having an infant born preterm [17, 18]. Our study shows the same result by using a Chinese population, and a smaller maternal pelvic size may be the underlying mechanism due to evolutionary adaptation.

Placenta previa is a risk factor for preterm birth [5]. The formation of the lower uterine segment and cervical dilation will cause a certain degree of spontaneous placental separation, which may result in severe haemorrhage and can indicate preterm birth [19]. As a clinical indicator of iatrogenic preterm birth, placenta previa accounts for 14.1% of all cases, which is much larger than the prevalence in China. To our knowledge, this is the first report of the morbidity of placenta previa in a large-scale, Chinese, scarred uterus population.

IPI is defined as the time from the most recent prior birth to conception of the index birth by Mckinney and his coworkers [20]. An IPI of 0 to < 12 months accounted for only a small portion of the data (*n* = 35,1.5%) because most of the women with IPIs less than 12 months were recommended for delivery of the baby. The 12 to < 24 months category was chosen as the reference group based on Mckinney’s study, and an IPI of 12 to < 24 months was associated with the lowest risk of preterm birth in both Mckinney’s and our studies.

Self-reported vaginal bleeding during pregnancy is predictive for preterm birth. The odds ratio of vaginal bleeding was 2.7 (95% Cl 2.03–3.70) in our study, with an incidence of 10.9%, smaller than that in other studies [21, 22]. Our study used number of vaginal bleeding during pregnancy instead of the presence of bleeding over the three trimesters or bleeding volume because we considered it to be easier for the patients to recall.

Women with a parity of less than two composed the majority of our data (*n* = 2171, 93.8%) and in the general population. This demographic characteristic is quite different in China due to the singleton policy that had been in place over the past years. Nulliparous and highly multiparous women are at higher risk of adverse pregnancy outcomes than those with low multiparity [23]. In our study, advancing parity showed a higher risk of adverse pregnancy outcomes. However, the nulliparity included in our study corresponded to women who had received myomectomy. Thus, there is no conflict between these two studies because of the different inclusion criteria.

Hypertension during pregnancy increases the risk of preterm birth. A recent meta-analysis including 55 studies found that women with chronic hypertension had high pooled incidences of preterm birth [24]. Preeclampsia was also found to be associated with high rates of preterm birth and puerperal complications, while gestational hypertension was only found to be related to preterm birth [25].

Apart from these six parameters, some factors were not included in our model and are thought to be important in the prediction of preterm birth. Age has always been considered a significant variable in preterm birth prediction. We have tried many ways to categorize age, including dividing it into three groups based on the report of a U-shaped relationship with preterm birth [26]. Sadly, none of these attempts give us a statistically significant result. Likewise, factors related to infection are important variables related to preterm birth. However, screening for infection requires sequential tests, including vaginal secretion cultures and inflammatory indexes, which are not fully covered by medical insurance in China. Therefore, factors related to infection were not included given their low cost-efficiency and data integrity.

After screening the above six factors among all the variables, a model was built and validated. The results of the discrimination and calibration tests are shown above. Overall, our model shows good discrimination and calibration. However, discrimination and calibration alone cannot capture the clinical consequences of a particular level of discrimination or degree of miscalibration [27–29]. To justify the practical applicability of our model, decision curve analysis was applied in this study. The results of the decision curve analysis indicate a worth-expecting practice in the clinic. With the threshold probability between 15 and 60%, the use of a nomogram in our study to predict iatrogenic preterm birth adds more benefit than either a treat-all-patients scheme or a treat-none scheme.

The distribution of medical resources in China is not even. Many hospitals in rural areas lack neonatal intensive care units, which leads to adverse outcomes for the newborns. Sometimes mothers with pregnancy complications cannot be treated effectively. Reasonable and efficient referral can improve this situation. Our model improves maternal and child outcomes by assessing the risk of iatrogenic premature birth in patients, thus assisting in referral-making and helping in the rational allocation of medical resources. A correct prediction can provide patients better medical care, thus improving the prognosis of the mother and foetus, while an incorrect prediction would waste local medical resources or delay the opportunity to treat patients. We recommend that doctors use lower cutoff values in districts with rich medical resources or high economic capability. Note that the applicable population for this model is the same as the inclusion criteria of this study, which means that this model is only suitable for pregnant women with more than 20 weeks of gestation. Meanwhile, new problems may arise at any time, so we recommend using this model sequentially during pregnancy.

### Strengths and limitations

The prediction model we developed is novel. To our knowledge, this is the first model to predict iatrogenic preterm birth in a scarred uterus population using the population from Northeast China. In our study, we selectively collected individual information that can be easily acquired during consultation or through some basic examinations. Thus, it is a convenient model that can be easily practised in the clinic for the recognition of high-risk populations and for making referrals, which means that it can be widely applied in primary health care institutions in rural areas of China that lack sufficient medical resources.

The shortcomings of this study are as follows: (1). The influence of maternal weight on preterm birth is complicated. We decided to collect all the data on weight and weight gain for all trimesters at first. Due to data loss, only weight before delivery was included in this study. (2). A total of 2315 cases were divided into a training set and a validation set; the nature of internal validation indicates one of the weakness of our study. (3). Due to the retrospective nature of this study, the data we collected, except for age and height, were all acquired before delivery. Therefore, the model is only valid for pregnant women with more than 20 gestational weeks.

## Conclusion

We built a model to predict iatrogenic preterm birth for pregnant women with scarred uteri. The variables included in the model were height, parity, IPI, vaginal bleeding during pregnancy, placenta previa, and hypertension during pregnancy. The nomogram we developed can assist doctors in evaluating the risk of iatrogenic preterm birth and deciding whether these patients should be referred to an higher tier medical centre; thus, better medical care can be provided to prevent adverse pregnancy outcomes and poor foetal conditions.

## Data Availability

The data analysed specifically for use in this study are not publicly available due to their use in ongoing research, but reasonable requests for data can be made to the corresponding author at the end of the research.

## Abbreviations

IPI: Interpregnancy interval
BMI: Body mass index
CI: Confidence interval
OR: Odds ratio
IQR: Interquartile range
AUC: Area under curve
IPTB: Iatrogenic preterm birth
GDM: Gestational diabetes mellitus
TOLAC: Trial of labour after caesarean
